# Setting-up a cross-border action-research project to control malaria in remote areas of the Amazon: describing the birth and milestones of a complex international project (Malakit)

**DOI:** 10.1186/s12936-021-03748-5

**Published:** 2021-05-11

**Authors:** Muriel Suzanne Galindo, Yann Lambert, Louise Mutricy, Laure Garancher, Jane Bordalo Miller, José Hermenegildo Gomes, Alice Sanna, Cassio Peterka, Hélène Hilderal, Hedley Cairo, Helene Hiwat, Mathieu Nacher, Martha Cecilia Suárez-Mutis, Stephen Vreden, Maylis Douine

**Affiliations:** 1 Centre d’Investigation Clinique Antilles-Guyane, Inserm 1424, Centre Hospitalier de Cayenne Andrée Rosemon, Cayenne, French Guiana; 2 The Ink Link, Paris, France; 3 DPAC Fronteira, Oiapoque, Brazil; 4 Agence Régionale de Santé de la Guyane, Cayenne, French Guiana; 5 National Malaria Control Programme, Ministry of Health of Brazil, Brasilia, Brazil; 6 National Malaria Control Programme, Ministry of Health of Suriname, Paramaribo, Suriname; 7 Laboratory of Parasitic Diseases, Institute Oswaldo Cruz/Fiocruz, Rio de Janeiro, Brazil; 8 Foundation for the Advancement of Scientific Research in Suriname, Paramaribo, Suriname

**Keywords:** Complex intervention, Action-research, Implementation science, Border malaria, Malaria control, Mining population, Neglected population, Mobile migrants

## Abstract

**Background:**

In French Guiana, gold miners working illegally represents a major reservoir of malaria. This mobile population, mainly of Brazilian descent, enters the French Guianese forest from neighbouring countries, Suriname and Brazil. A complex and innovative intervention was piloted as a cooperation with the three involved countries involved to control malaria in this specific population. The principle was that health workers called “facilitators” provide the participants with a self-diagnosis and self-treatment kit along with adequate training and material to rapidly manage an episode of malaria symptoms on their own, when they find themselves isolated from health care services.

**Methods:**

This paper describes the design, development, content of the intervention and players’ organization of this multi-country project, the opportunities and constraints encountered, and the lessons learnt at this stage.

**Results:**

The choice not to implement the usual “Test and Treat” approach within the community is mainly driven by regulatory reasons. The content of medical messages tends to balance the tension between thoroughness, accuracy and efficacy. The wide range of tools developed through a participatory approach was intended to cope with the challenges of the literacy level of the target population. Despite the difficulties encountered due to language, regulation differences and distance between partners, cooperation was fruitful, due to the complementary of stakeholders, their involvement at all important stages and regular face-to-face meetings.

**Discussion and conclusion:**

This experience shows the feasibility of an ambitious project of action-research in a border malaria context, involving several countries and with a mobile and undocumented population. It reveals some factors of success which may be transferable in analogous settings.

**Supplementary Information:**

The online version contains supplementary material available at 10.1186/s12936-021-03748-5.

## Background

**Fig. 1:**
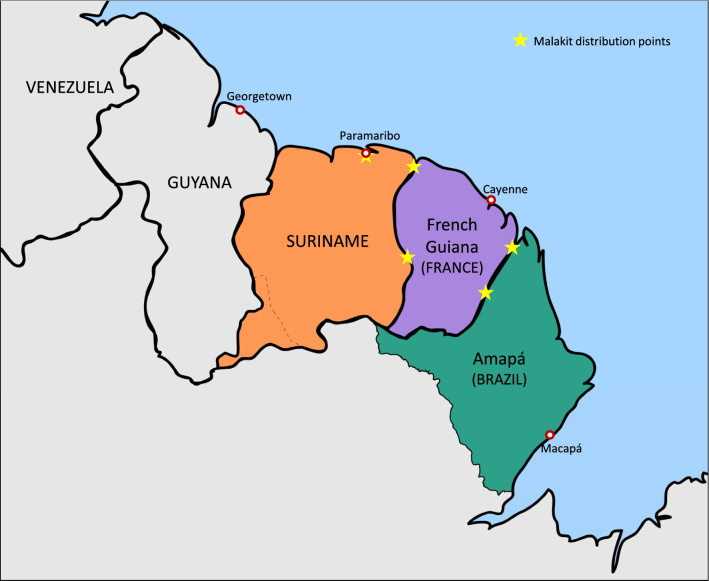
Map of the Guiana Shield with the Malakit distribution points

The Guiana Shield is a Region shared by French Guiana, Suriname, Guyana and some parts of Venezuela, Colombia and Brazil (Fig. 1). The soil, rich in gold and other minerals, is predominantly covered by dense Amazonian rainforest and is the source of livelihood of various groups, among which gold miners. Malaria is endemic in this region. Although the number of cases is decreasing, malaria transmission is still intense within the forest. The impact of illegal gold mining activity on the transmission of malaria is now well documented, and the mobile population involved has been identified as a key malaria reservoir. Gold miners in French Guiana are mostly of Brazilian origin and arrive in the country from Brazil or Suriname. They are estimated to be 10,000, with a median age of 37 years [interquartile range (IQR) 30–45] and a male predominance (70.6%), according to the Orpal study undertaken in 2015 [1–6]. The three countries directly affected by this intense population flow have initiated a collaborative reflection to address this problem, despite their contextual differences, detailed in Additional file 1.

**Fig. 2:**
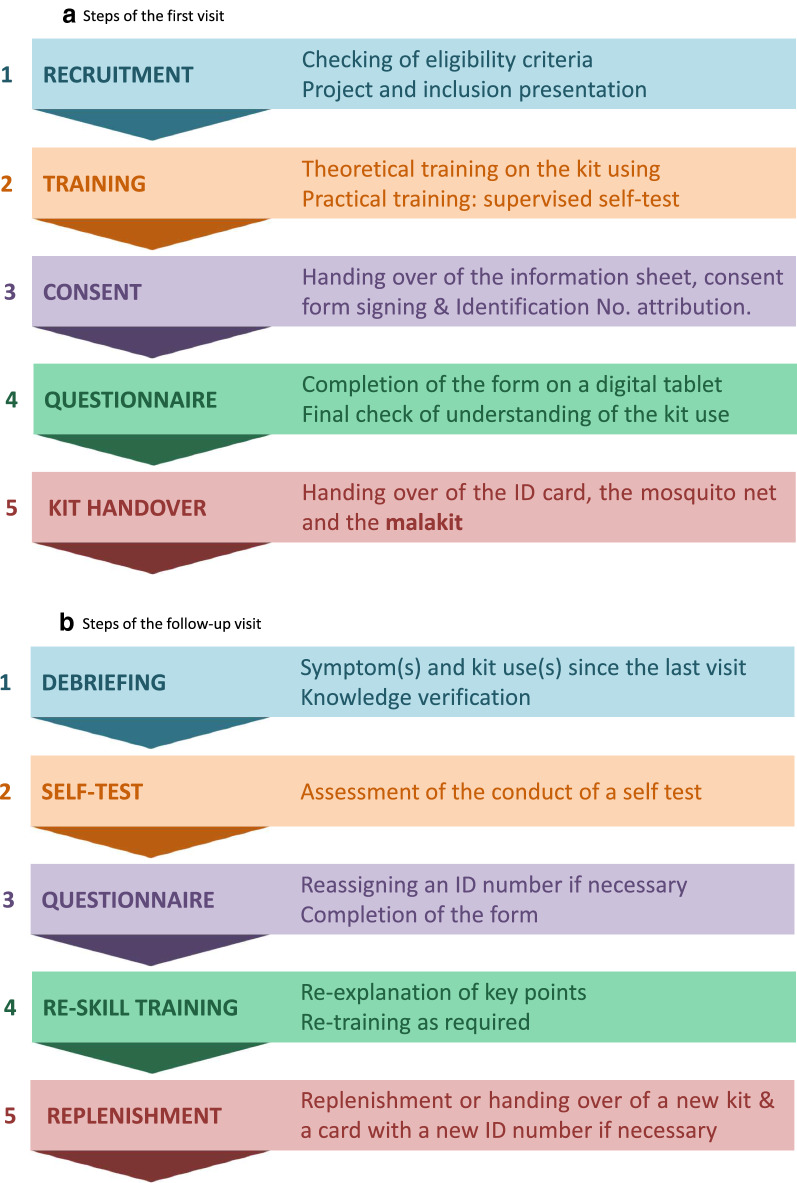
Steps of the visits. **a** Steps of the first visit. **b** Steps of the follow-up visit

This cooperation resulted in the implementation of ‘Malakit’, an innovative pilot project, aiming at evaluating a new strategy to control malaria among migrant gold miners working illegally in French Guiana. The principle was to provide participants with a self-diagnosis and self-treatment kit along with adequate resources to handle rapidly and adequately an episode of malaria symptoms by themselves, when isolated in the Amazon forest [3]. It was a prospective multicentre research project which can be classified as a complex intervention study [7]. Outcome evaluation was based on pre-post evaluation and longitudinal follow-up, i.e. collection of data by Malakit facilitators during inclusion visits when participants receive their first kit, and during subsequent visits after they have experienced the use of the kit and/or episodes of malaria symptoms (Fig. 2). Because border malaria is a problem historically associated with anti-malarial resistance on different continents, the various interventions attempting to tackle the problem in a given context may or may not be transferable to other contexts [8, 9]. In order to facilitate the potential transfer in other contexts, a detailed description and the learnings from the setting up experience of the intervention is provided here.

## Methods and scope of the article

Despite the emergence of specific reporting guidelines for complex non-pharmacological interventions, descriptions of interventions are still insufficient and inadequate. One of the objectives of this article is to address the following question, as recommended by the UK Medical Research Council (MRC) [7]: *Can you describe the intervention fully, so that it can be implemented properly for the purposes of your evaluation, and replicated by others?*

Although, the protocol of this study was published in 2018 [3], it seems necessary in this particular context, to explain the underlying logic of the design, development and deployment phases, how the whole project was structured, the role and responsibilities of the multi-countries players, the challenges and opportunities of the cross-border context in terms of cooperation. The findings are based on self-criticism feedback collected from the players involved in the setup and implementation of the study. The kit and its contents, the training of field actors, the communication tools and the content of the messages are described to aid the reproduction of the intervention in similar contexts and constitute a first step for forthcoming implementation research. Fidelity and lessons learnt from confronting the underlying hypotheses of the intervention with its actual implementation on the field will be explored in another article.

## Results

### Intervention development steps

**Fig. 3:**
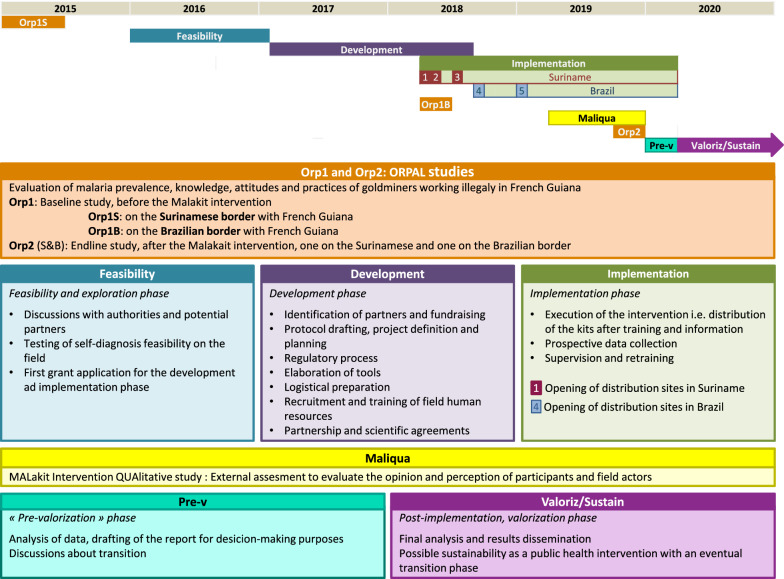
Phases of the Malakit project over time

The intervention was designed after the initial idea was validated by the partners from the three countries involved i.e. the distribution at border resting sites of a self-diagnostic and self-treatment kit to gold miners, to be used when needed, within the context of their activity on the French Guianese territory [3]. The design of the intervention itself overlaps with that of its evaluation (Fig. 3).

#### Intervention sites

The choice not to develop a usual community-based approach as implemented in several places in the world with great success, such as in Suriname, is mainly for regulatory reasons [10]. In French Guiana, a decree allows staff of Delocalized Centres for Prevention and Care to perform Rapid Diagnostic Test for malaria (RDTs) after a specific training and only medical doctors are authorized to prescribe drugs [11]. Mobile health units are not an option either because of the lack of health professionals in French Guiana and geographical and safety constraints in the forest [3]. The territory is vast, the number of mining camps is high—around 600 at the end of 2017 [12]—and the access to sites sometimes is very arduous, and/or, illegal without authorization.

Illegal gold mining is well organized; each person has a role to play. Social life is also structured and has its own codes. However, it is not a proper pyramidal organization and communities are transient, due to the absence of a permanent commitment to bosses and to the great mobility of many *garimpeiros* [13]. Even with regulatory exceptions specific to the territory, it would have been a huge challenge to identify, train and supervise members of the community to be intermediaries, especially in the most isolated areas. Hence, the intervention was planned for implementation where the study population could be reached i.e. on rear bases on the border rivers in Suriname and Brazil, where goldminers rest before or after going to French Guianese gold mining sites. Before the intervention, the Orpal study revealed that *garimpeiros* were accessible, available, and willing to give time for health studies, and more specifically, to answer questions about their activity and their health [6]. Complementary to routine malaria care offered at the same place, the approach was to provide gold miners with the material and resources required to handle by themselves an episode of malaria symptoms, once in the forest.

#### Kit development

It was decided, in consensus with partners, that the main objective would be to increase the use of an appropriate and complete malaria treatment against *Plasmodium falciparum,* the most common among *garimpeiros* at this period [6], including artemisinin-based combinations, which are also efficient for *Plasmodium vivax* malaria attacks (but not to prevent relapses). The ultimate goal was to prevent the emergence of artemisinin-resistant *Plasmodium falciparum* parasites by addressing the risks associated with the practices of the study population.

##### Diagnostic test

The strategy of training key persons within a community to perform diagnostic tests to other people has been widely used in low income countries. Despite heterogeneity of efficacy and cost-effectiveness after scaling-up, the feasibility of training laypersons to perform RDT, even with a low education level, has already been demonstrated [14–16].

One innovative aspect of Malakit is the self-performed RDT test in case of malaria symptoms, which has only been implemented previously in research among travellers. Study designs varied with diverse types of RDTs and with instruction methods ranging from simple written instructions to actual training. Conclusions of these studies have a limited transferability due to the specific contexts, such as the possibility to consult a health professional, the risk of false negative inherent to the type of test, or the limitations of the study design [17–21]. However, one study testing a malaria prevention programme for oilfield service employees, relying on a kit with self-tests and curative medication, concluded by stressing the key role of the instructions [18].

The Malakit intervention hypothesized that with adapted directives and prior supervised practice of self-RDT, gold miners could then perform a self-RDT, whatever their literacy level, even while in the forest with malaria symptoms. The Access Bio’s CareStart™ Malaria pLDH (Pan) can detect (but not distinguish) the four main species of human malaria parasites (*P. falciparum*, *P. vivax*, *Plasmodium ovale* and *Plasmodium malariae*), is easy to read (two-band test) and can withstand storage at temperatures up to 40 °C (mean temperature range in the Amazon being 25 to 27 °C); it was thus the best RDT candidate [3]. However, it was not available as a single kit, but the company agreed to produce individual packaging and to provide lancets with a retractable needle to avoid blood exposure.

Three tests were included in the Malakit in order to allow for different scenarios before a miner returns to a distribution site for a new supply: having several episodes of malaria symptoms, an invalid test result, or giving a test to someone else.

##### Treatment

The selected artemisinin-based combination therapy uses artemether 20 mg/lumefantrine 120 mg (Coartem®), and is the one recommended by the respective health authorities in the three countries involved in the project, in accordance with World Health Organization (WHO) recommendations [22].

A single dose of primaquine is recommended for its gametocytocidal effect in addition to treatment regimens [23]. To this end, two pills of 7.5 mg primaquine were included in the kit to be taken along with the first intake of Coartem®. The addition of paracetamol (one blister of ten 500 mg tablets) comes from the idea that in case of a negative RDT, the person cannot be left without any alternative to taking anti-malarial treatment. The corresponding instruction was to use it to relieve pain and fever, whatever the result of the test.

##### Other features of the kit

The desired characteristics of the container of the kit were to be waterproof, resistant and easily transportable to fit gold miners’ environment, and to contain sufficient information to decrease the risk of misuse in case someone obtained it without training. Around 40 gold miners were shown several design options on the Surinamese border and contributed to the creation of the final prototype.

The container of the kit was made of plastic, wallet-sized (i.e. 20 by 15 cm) (Additional file 2), and had a hole for a carabiner. It was split into two pockets (Additional file 3): in the first one, the holder of the RDTs (Additional file 4) and in the second one, the holder of the treatment (Additional file 5). The illustrated instructions in Portuguese were directly printed on the two plastic holders.

A small waste bag with a biological hazard symbol was added to the kit so the participants could carry and bring back safely and handily the wastes of the used RDT (Additional file 2).

A long-lasting insecticidal net (LLIN) for hammocks completed the kit.

#### Training of facilitators

The function of the facilitators, main protagonists of the intervention, was to carry out inclusion and follow-up visits as described in Fig. 2. National or local malaria programme managers provided general training about malaria to the facilitators of their respective country. The facilitators then received a theoretical training on their upcoming duties (Additional file 6), with the corresponding tools (Additional file 7). Finally, practical training on the field completed the curriculum, which proved a more efficient way of learning for facilitators.

The contents of the theoretical training could not be covered in a single session. Experience showed that it was much more successful to split the training into two parts. In the first part, the objective was to become proficient in performing a first visit (training, enrolment and data collection). The second part focused on mastering data collection during follow-up visits, which implies debriefing the participants about their episode(s) of symptoms, use(s) of the kit and movements since the last visit on a distribution site. Due to administrative (obtaining of visa) and logistical constraints, it was not possible to gather all facilitators and supervisors for one training session. Therefore, three sessions were provided, which were refined and improved over time. Simulations and confrontations with the reality in the field during the first training allowed optimizing the structure of the inclusion process. Smaller audiences also enabled to address partially the issue of language differences and heterogeneity of background and education levels.

#### Information, education and communication (IEC) content and tools

All messages are presented in the Table 1.

**Table 1:** Information, education and communication tools and content

Tool	Medium	Objectives	Content of messages
Animated video (Additional file 9)	Displayed by the facilitators during the training Shared with participants owning a smartphone, for further sharing on social networks Included in the Malakit app distributed to participants	Presentation of the malakit (object) and general presentation of the project	Kit to detect and treat malaria in case of symptoms Content of the kit: “3 RDTs and full malaria medication” Importance of taking the entire malaria medication and risk of resistance to ACT Kit given for free Location of distribution sites
Illustrations and text about the RDT	Printed on the plastic RDT holder of the malakit Displayed by the facilitators during the training	Instructions for performing a self-RDT	“*When you feel sick, the best is to visit in a health center*” “*Be careful when the test is negative, do not use Coartem®*” “*Take paracetamol if you feel fever or pain*” Visual explanations on how to interpret the result (two lines = positive, one line in front of C = negative etc.) “*In case of positive RDT, take the treatment against malaria*” “*In case of negative RDT, do not take the treatment against malaria*” “*In case of invalid RDT, redo the test*”
Illustrations and text about the treatment	Printed on the plastic Treatment holder of the malakit Displayed by the facilitators during the training	Explanations and instructions on when and how to take the different drugs of the kit	“*If you do not feel better, try to consult a health center*” “*Be careful when the test is negative, do not use Coartem®*” Paracetamol is to be taken in case of fever or pain (1000 mg, 3 times a day maximum) “*If the result of the malaria test is positive, do the entire treatment*” Primaquine is not allowed for pregnant women “*Take 2 pills of primaquine with the Coartem®*” Coartem®: “*Take 4 pills in the morning and 4 pills in the evening during 3 days*” Importance of taking the entire malaria medication to eliminate the parasite, even if the fever disappears and you feel better
RDT video (Additional file 8)	Displayed by the facilitators during the training Shared with participants owning a smartphone Included in the Malakit app distributed to participants	Instructions for performing a self-RDT	Explanations on how to interpret the result In case of invalid RDT, redo the test If the test line is clear and indefinite, consider the test as positive The RDT waste should be disposed in the yellow bag and given to the facilitators
Illustrated posters (Additional file 10)	Displayed and explained by the facilitators during training	Support tool for the training dispensed by facilitators to impart knowledge about malaria and its treatment and introduce the explanation about when and how to use correctly the kit	*Poster “One infection–one appropriate treatment*”: importance of knowing the cause of the infection. The treatment of the kit will not work if the cause of the symptoms is not malaria *Poster of parasites*: If the treatment is not complete, the strongest parasites will remain, and with time the treatment will lose its effect on malaria *Poster* “*Malaria parasite*”: existence of *P. vivax* and *P. falciparum*. The RDT of the kit detects both. The treatment of the kit eliminates the parasite circulating in the body for both types and therefore, eliminates symptoms. However, if symptoms reappear one month later, it can be *P. vivax* which was dormant in the liver and is now waking up. In this case, it is important to consult a health professional *Poster of risky situations* (include severe signs and the presence of a heart condition): Beware, in your case, malaria can be severe. Start the treatment and go to a health center as soon as possible
Information video	Mobile application	Presentation of the disease	Explanations on: vector, parasite, transmission, symptoms, diagnostic methods
Prevention video	Mobile application	Presentation of the prevention measures against malaria	Risk of mosquito bites is higher during the night Use of mosquito nets Use of repellent on the skin Advice to avoid places where mosquitoes can breed (avoid standing water, keep inside and outside clean
Interactive module	Mobile application	Personal tutorial for malakit use in case of symptoms	“*You should consult a medical doctor in a health center*” “*The malakit is not suitable for people under 15 year old*” List of malaria symptoms (with pictograms) Visual explanations on how to interpret the result of the RDT List of risky situations which includes signs of severe malaria and a history of heart condition (with pictograms) In case of signs of severe malaria: “*Beware*, *in your case, malaria can be severe. Start the treatment and go to a health center as soon as possible*.” “*Beware, if you are pregnant or breastfeeding, do not take primaquine. Go to a health center as soon as possible.*” Treatment instructions Reminder alerts for each intake of Coartem®, and a question after three days to confirm whether the medication was taken entirely “*Sleep under a mosquito net to avoid malaria transmission.*” “*You should consult a health center for a medical opinion*” if the person does not feel better after the end of a treatment In case of a negative test: “*It is not recommended to take the treatment against malaria without positive test. You can take paracetamol in case of fever. If symptoms persist, do another test and consult a health center*”

The text in italics refer to all written messages

##### Medical messages

Some specific points of the intervention required the consultation of experts in order to choose between different options:

- Completeness of the treatment:
  Interrogation between the two following message options: “*Take your medication entirely if you have a positive RDT”* or *“If you start treatment, you have to finish*”, considering that the person may take the medication with a negative diagnosis. The first option was agreed upon in order not to give too many messages and to avoid the suggestion that it is correct to take the medication without a positive diagnosis of malaria.
- Message to avoid risk of overdose for children:
  The question was either to indicate a dosage according to weight and age or to state that the kit is only for adults (over 15 years old). Eventually the main message was that the kit is for personal use only, and participants with symptomatic children were specifically advised to consult a health professional.
- Indication for paracetamol medication:
  The two options were: “*Take paracetamol when the RDT is negative*” as opposed to Coartem® intake in case of positive RDT, and “*Take paracetamol when you feel fever or pain/ache; paracetamol is the sole medication you can take if the RDT is negative*”. The second option was selected since paracetamol can be regardless of the outcome of the RDT, and to stress on the importance of positivity for malaria treatment.
- Contraindications:
  The risk–benefit balance was finally in favour of the absence of interdiction of taking the antimalarial medication considering that it is very tricky at a personal level to identify contraindications. The contraindication of cardiac history for Coartem® was addressed by giving the following message: “*If you take medication for the heart, start the malaria treatment as soon as you have a positive test but also seek medical care as soon as possible*.” As described in Table 1, the same advice is given in case of situations at risk. Finally, pregnant and lactating women were warned that they should not take primaquine in any circumstances.
- Instructions regarding possible relapses of *P. vivax:*
  In order to avoid giving instructions that would probably not be followed, it was decided to recommend consulting a health centre only in case of suspicion of relapse of *P. vivax* and in case of persistence of symptoms. Indeed, recommending to use the malakit as a first-aid kit and then to go to a health centre for a professional opinion and specific malaria diagnosis did not seem relevant, especially if the person feels better after using a kit.

##### Instructions printed on the kits

During the feasibility assessment phase (Fig. 3), an artist developed illustrated instructions for self-RDT use through a participatory method with participants from the community on the Maroni River side [24]. The outcomes of this strategy were encouraging enough to go further. During the development phase, the project team supported by the local association helped the illustrator to better adapt these instructions, by testing them on the Oyapock River side: *garimpeiros* were asked to perform a self-RDT with only drawings as explanation. This allowed for example to determine the need to number the images to indicate the right order in which the images are read. Participants were also involved in adjusting the instructions of medication use to their literacy by changing the level and amount of information, disposition of images and text. For example, thanks to their contribution, pictures were added to indicate the time of intake of Coartem®, i.e. a sun for the morning and a moon for the evening (Additional file 5). All the illustrated instructions were tested from a paper print. Once printed on the plastic pouches, the readability seemed to be reduced, despite excellent print quality.

##### Videos

A similar participatory approach was used for the development of the tutorial video to perform a self-test (Additional file 8). The content of both tools—illustrated instructions and video—was harmonized to be concordant. An animated video with a humorous scenario was produced to promote the intervention toward the study population (Additional file 9). It was used by the facilitators to present the project to participants before the actual training and could be shared by the participants from their phone to other members of the community.

##### Smartphone application

A smartphone application was developed since a widespread use of smartphone was observed in the community. It consisted of several modules of information including the illustrated instructions and the videos described above, but also other animated videos on malaria and an interactive module to guide the participant on kit usage.

### Coordination and cooperation

#### Description of the organization

**Fig. 4:**
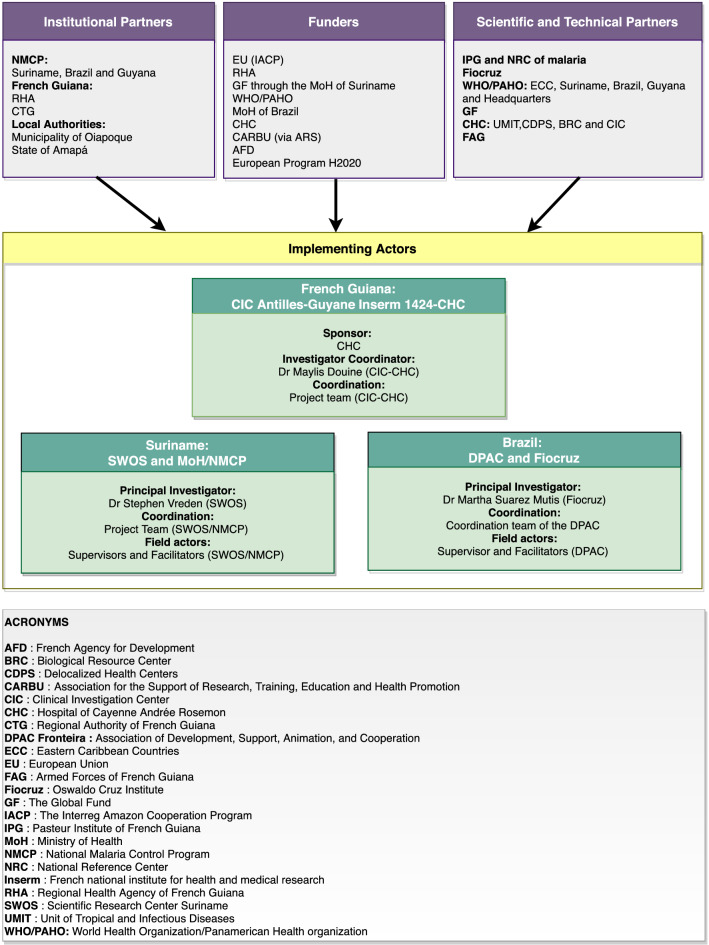
Organizational chart of the Malakit project

As an intervention research project [3], the Clinical Investigation Centre Antilles-Guyane (CIC-AG), a clinical research department attached to the Hospital of Cayenne (CHC) took the lead of the project. The sponsor of the study was the CHC and the sponsor investigator was a medical doctor of the CIC-AG (Fig. 4).

The CIC-AG project team was responsible for (1) assessing feasibility, (2) drafting the protocol and all study tools, (3) seeking funds and managing the bulk of the project funds, (4) searching for investigation centres, (5) searching for technical, scientific and institutional partnerships and agreements drafting, (6) informing and training of the persons in charge of the investigation, (7) contributing to the logistical implementation of the study, (8) developing, implementing and managing the information system, (9) supervising the study intervention delivery, (10) analysing data and disseminating results and (11) communicating about the project.

The CIC-AG’s promotion department was in charge of the clinical trial monitoring of the study with a dedicated clinical research associate. The strategy followed the recommendations of the Orpal study [6], which was previously conducted by the CIC-AG. Close links between intervention and evaluation in terms of design led to a unique coordination structure. The cross-border context required cooperation with neighbouring country-organizations on the field. Therefore, partner structures were selected according to their resources and experience in social and health mediation with the community of study and their legitimacy and ability to recruit participants outside French Guiana.

##### In Suriname

The National Malaria Control Programme (NMCP) has a strong experience in health promotion and primary healthcare among gold miners on the field [25]. The Foundation for the Advancement of Scientific Research in Suriname (SWOS) is a research foundation partnering with the University of Suriname and the Academic Hospital of Paramaribo (AZP). It has experience in research studies and has administrative and logistical resources to implement complex interventions. The chair of the SWOS was the principal investigator and the coordinator of Surinamese study sites in the Malakit project. The SWOS, in conjunction with the NMCP, was in charge of the recruitment of the field human resources, on-site project management (including logistics) of the study onsite and also contributed at communicating about the project.

##### In Brazil

The French Guianese border is far from the centres of Brasilia and Rio de Janeiro and from Macapá (capital of Amapá state) but both central and local authorities were involved as much as possible at all stages of project development. A local non-government organization (NGO), DPAC fronteira (or “DPAC”), with a history of social and health mediation with the study population was chosen to implement the project. DPAC also had experience in French-Brazilian international cooperation. They were in charge of staff recruitment and management of the Brazilian study onsite. The principal investigator and scientific counterpart in Brazil was a researcher working on malaria epidemiology at the Foundation Oswaldo Cruz (Fiocruz) in Rio de Janeiro in order to complement scientifically the DPAC field of competence (Fig. 4).

The responsibility of the realization of the endline study Orpal was given to the Fiocruz along the Brazilian border and to the CIC-AG along the Surinamese border. Key considerations for successful cooperation between countries were (1) the formulation of a convergent goal and joint efforts to achieve it, (2) solicitation and involvement of all stakeholders at each important phase of the project, (3) fruitful interactions between institutions, (4) scientific structures and social associations in the field, (5) the consistency of the main players in each country.

#### Communication/information sharing

The language of the study population and of the facilitators was Brazilian Portuguese, however, other actors spoke French, English, or Dutch. The main protagonists of the project were either located in Paramaribo, Cayenne, Rio de Janeiro or along the Maroni and Oyapock border rivers. The distance and transport constraints, and the linguistic differences were the main constraints for communication, which is the key to successful project management. Providing enough time, person-time and funds for sufficient bilateral and international meetings, and planning the systematic presence of professional interpreters during trainings and meetings, are factors identified from this experience to enhance better communication between stakeholders. Furthermore, digital tools can be useful for advancement between two face-to-face meetings but imply allowing time for training of players to ensure commitment to the tool.

#### Regulatory and administrative aspects of the study

In France, the sponsor of a study is responsible for ethical clearance in countries where participants are included, i.e*.* Brazil and Suriname. In Brazil, the organization of the principal investigator, Fiocruz, conducted the translation and the submission of the protocol internally and to the Brazilian national committee. In Suriname, ethical clearance submission with the national committee was also delegated to the Surinamese principal investigator. Study insurance for each country was contracted by the sponsor. Differences in what constitutes good clinical practices were identified between countries in the course of the project, but adaptation efforts were made on each side. For example, those responsible for the investigation of both countries (Suriname and Brazil) contributed well to the monitoring carried out by the research associate. A better prior knowledge of the regulations in each country can probably save time. Administrative procedures such as the registration of health products with Brazil’s National Sanitary Regulatory Authority and Genetic heritage System, customs formalities or visa applications were barriers to smooth cooperation. A letter of intention between all countries at higher administrative levels may be a solution to these issues.

#### Logistics

The supply chain management for this project was a complicated and time-consuming task due to delays in transferring funds, absence of customs at the border between French Guiana and Brazil which has made the transportation of materials across borders more complex, long and complicated procedures for donations of medical products and shipping mistakes from provider. The timing of such an international initiative should take into account these possible problems.

#### Participants safety

The Surinamese coordination suggested two safety measures to protect the participants, i.e. the implementation of a participants advocacy and the assembling of a Data Safety Monitoring Board (DSMB). For the first one, the Surinamese coordination partnered with the Brazilian embassy that made available a hotline number for Malakit participants. Each participant was told during inclusion that a person independent of the project could be contacted if he/she had any concerns or questions that could not be shared with the people of the project, such as an inappropriate behaviour of a facilitator.

For the DSMB, the sponsor contacted four malaria experts around the world who had access to safety information such as reports of the RDT quality control, side effects or misuse indicators. They gathered twice to discuss on this information to give recommendations and ask for clarification when needed.

## Discussion

This article presents the development of the Malakit intervention, opportunities, constraints and lessons learnt at this stage of the project. Despite a complex international context implying distance working, cultural and language variety, regulation barriers, and a sensitive and mobile target population, this unprecedented project was implemented in a rather short period of time i.e. about one year and a half between the initial idea and the first Malakit distribution. Some of the ingredients underlying the successful setup of the project are (1) the complementarity of stakeholders i.e. health professionals, public health specialists, research clinical specialists, information system specialists, malaria experts, illustrator, health and social mediation specialists, actors in contact with the study population and community representatives, (2) the consideration of feasibility information, (3) the participatory approach in developing a wide range of complementary materials and tools adapted to the community, and (4) good external communication on the project to all partners, health institutions and the scientific community. This experience confirms that reduction of the administrative procedures, such as visa and custom formalities can smoothen efficient implementation of activities in border areas, as reported in literature review [26].

Uncontrolled land borders have been identified as a risk factor for the re-introduction of malaria since the 1960s [9, 27]. Border malaria as defined by the WHO Evidence Review Group can be found on almost all continents [26], countries involved are however at varying stages of collaboration. While for instance Bhutan and India hold a meeting late November 2019 to develop a roadmap for cross-border collaboration for the next three years [28], the Trans-Kunene Malaria Initiative has already disseminated results on their joint program at the Angolan–Namibian border [29] and the binational cooperation between Haiti and Dominican Republic won the Malaria Champion award for its remarkable achievements in 2017 [30]. Many known contributors to border malaria [26] were observed in the context of the Malakit project, such as remoteness, forested area, mobility across borders, illegal status of the population of interest, insufficient surveillance in this particular population, behaviour fostering the emergence of resistance, security issues, differences of malaria policies between countries, cultural and language differences and heterogeneity of malaria transmission. The particularity of malaria in French Guiana is that it is not limited to border areas, where are located the health posts, but also in the most remote places in the interior of the territory. A joint project, or study might aid cross-border collaboration [26, 31]. The survey carried out by Laos and Vietnam in 2010 is a good example. From a population-based and vector-based study on malaria, several achievements in cooperation have emerged, i.e. regular exchange of malaria surveillance data and the joint implementation of a round of Indoor Residual Spraying—already carried out in Vietnam—in the villages of Laos with high malaria prevalence [31]. The Malakit project has as well led to a better mutual knowledge of the stakeholders and their respective functioning and to the continuous improvement of communication and information sharing. In this way, it contributed to the strengthening of cooperation capabilities.

## Conclusion

This initiative shows that an action research project with an innovative approach involving three countries concerned by border malaria is possible despite many challenges. Such a project can also stimulate further cooperation and may provide a model to be transferred to other regions with similar contexts.

## Supplementary Information

**Additional file 1.** Contextual differences between countries involves in the Malakit project. The malaria and gold mining context of French Guiana, Suriname and Brazil are described here.

**Additional file 2.** Malakit pouch. Picture of a malakit closed.

**Additional file 3.** Malakit pouch opened. Picture of a malakit opened.

**Additional file 4.** RDT pocket of the malakit. Picture of the RDT pocket heads (illustrated instructions) and tails.

**Additional file 5.** Treatment pocket of the malakit. Picture of the Treatment pocket heads (illustrated instructions) and tails.

**Additional file 6.** Malakit training course. Content of the training.

**Additional file 7.** Tools and contents for facilitator training. The different tools and their content used to train the facilitators in their tasks.

**Additional file 8.** RDT video. A tutorial video to perform a self-diagnostic test with a voice-over in Portuguese.

**Additional file 9.** Animated video. A teaser video which presents the Malakit project.

**Additional file 10.** Training posters. The drawings used by the facilitators to train the Malakit participants.

## Data Availability

All data generated or analysed during this study are included in this published article and its Additional files.
