# Supplementary material for: Setting-up a cross-border action-research project to control malaria in remote areas of the Amazon: describing the birth and milestones of a complex international project (Malakit)
Source: Malar J. 2021 May 11;20:216. doi: 10.1186/s12936-021-03748-5 (PMC8111981; doi:10.1186/s12936-021-03748-5)
Supplement: Supplementary file 1 — Additional file 1. Contextual differences between countries involves in the Malakit project. The malaria and gold mining context of French Guiana, Suriname and Brazil are described here. [file 12936_2021_3748_MOESM1_ESM.docx]

**Contextual differences between countries involved in the Malakit project**

French Guiana is a French overseas territory in South America. The Regional Health Agency (RHA), representing the French Ministry of Health, can develop local policies, but must comply with French and European legislation. Gold mining without a mining license is fought by the French military forces on the territory through “Harpie” operations [1]. Traveling conditions and access to mining camps are particularly difficult in the French Guianese primary forest. In turn, health services are hard to reach for gold miners, and their illegal status make them reluctant to attend. The incidence of malaria in French Guiana increased during the 1990s and 2000s, with a peak at 4,479 cases in 2005. The number of cases then progressively dropped to 258 cases in 2016 [2,3]. The deployment of ACT use, impregnated nets, “Harpie” operations and positive effects of the malaria programs of neighboring countries were probable contributing factors to this decrease. However, cross-referencing information suggest a different situation in the interior of the country covered by rainforest. [3–6]. A study assessing the epidemic situation in the illegal gold mines (Orpal study) carried out in 2015 on the border with Suriname among gold miners working in French Guiana, estimated the prevalence of *Plasmodium* carriers at 22.3% (CI=[18.3-26.3], n=94/421), up to 46.8% in some places, with a majority of *Plasmodium falciparum* (58% including the co-infections)[7].

Suriname is a middle income country, scarcely populated, where the activity of gold mining is either allowed or tolerated, even when undertaken by migrants. The country has seen great progress in the fight against malaria during the past decades. With the support of international organizations such as the Pan American Health Organization (PAHO) and the Global Fund to fight AIDS, Tuberculosis and Malaria, the country has reached a near-elimination level through tailored strategies. The proportion of imported cases in 2017 was 92.6% (498), among which 95.6% from French Guiana, which makes the problem of malaria among migrant gold miners a major issue for the National Malaria Control Program (NMCP) [8–11].

Brazil accounts for a quarter of malaria cases in the Americas in 2018. More than 99% of malaria cases occur in the Amazon Region. The Brazil-French Guiana border is the largest border of France but the second smallest of Brazil. Over 90% of the garimpeiros (in Portuguese small-scale and/or illegal gold miners) working in French Guiana is born in Brazil but compared to the 200 million inhabitants in Brazil, this cross-border flow seems negligible [12]. Only 4% of the malaria cases were imported in the country in 2018, among which, 7% from French Guiana, in third position after Venezuela and Guyana [13]. Despite a decentralized management of health care, local health authorities report to the NMCP in Brasilia and to the State government in Macapá. Decision-making was complicated by this multi-layered organization, and by the political transition following the presidential elections in 2018.

[1] Ministry of Armed Forces 2017 - 2020. Point de situation des opérations du 15 février 2018 n.d. https://www.defense.gouv.fr/english/actualites/operations/point-de-situation-des-operations-du-15-fevrier-2018 (accessed January 21, 2020).

[2] Ardillon V, Eltges F, Chocho A, Chantilly S, Carvalho L, Flamand C, et al. Evolution de la situation épidémiologique du paludisme en Guyane de 2005 à 2011. Bulletin de Veille Sanitaire - Cire Antilles-Guyane 2012;1–2:5–11.

[3] Andrieu A, Ardillon V, Carvalho L, Petit-Sinturel M, Quet F, Bourdillon F. Surveillance du paludisme. Bulletin périodique : novembre 2017 à janvier 2018. Le Point Épidémiologique CIRE Guyane 2017;N°1/2018:3.

[4] Pommier de Santi V, Dia A, Adde A, Hyvert G, Galant J, Mazevet M, et al. Malaria in French Guiana linked to illegal gold mining. Emerg Infect Dis 2016. https://doi.org/10.3201/eid2202.151292.

[5] Mosnier E, Carvalho L, Mahamat A, Chappert JL, Ledrans M, Ville M, et al. Épidémies multiples dans des camps d’orpaillage en forêt amazonienne (Guyane française) en 2013 : quelles leçons pour l’accès aux soins et à la prévention ? 2015:181–9.

[6] Pommier de Santi V, Djossou FCL, Barthes N, Bogreau HC, Hyvert G, Nguyen C, et al. Malaria Hyperendemicity and Risk for Artemisinin Resistance among Illegal Gold Miners, French Guiana. Emerging Infect Dis 2016;22:903–6. https://doi.org/10.3201/eid2205.151957.

[7] Douine M, Musset L, Corlin F, Pelleau S, Pasquier J, Mutricy L, et al. Prevalence of Plasmodium spp. in illegal gold miners in French Guiana in 2015: a hidden but critical malaria reservoir. Malar J 2016;15:315. https://doi.org/10.1186/s12936-016-1367-6.

[8] Heemskerk M, Duijves C. Study on the knowledge, attitudes and practices of malaria and malaria treatment in the small-scale gold mining sector in Suriname. Submitted to the US Agency for International Development by the Systems for Improved Access to Pharmaceuticals and Services (SIAPS) Program.; 2013.

[9] Hiwat H, Martínez-López B, Cairo H, Hardjopawiro L, Boerleider A, Duarte EC, et al. Malaria epidemiology in Suriname from 2000 to 2016: trends, opportunities and challenges for elimination. Malar J 2018;17. https://doi.org/10.1186/s12936-018-2570-4.

[10] van Eer ED, Bretas G, Hiwat H. Decreased endemic malaria in Suriname: moving towards elimination. Malar J 2018;17:56. https://doi.org/10.1186/s12936-018-2204-x.

[11] Ministry of Health Malaria Program. Annual report 2016. Paramaribo, Suriname: 2017.

[12] Suárez-Mutis MC, Martinez-Espinosa FE. Malária. In: Coura JR & Pereira NG. Fundamentos das Doenças Infecciosas e Parasitárias. Elsevier. Rio de Janeiro: 2019.

[13] Ministério da Saúde, Secretaria de Vigilância em Saúde, Departamento de Vigilância de Doenças Transmissíveis, Coordenação-Geral dos Programas Nacionais de Controle e Prevenção da, Malária e das Doenças Transmitidas Pelo Aedes. Boletim epidemiológico de malária nas fronteiras e importada de outros países. 2019.
