# Supplementary figures and images for: Setting-up a cross-border action-research project to control malaria in remote areas of the Amazon: describing the birth and milestones of a complex international project (Malakit)
Source: Malar J. 2021 May 11;20:216. doi: 10.1186/s12936-021-03748-5 (PMC8111981; doi:10.1186/s12936-021-03748-5)

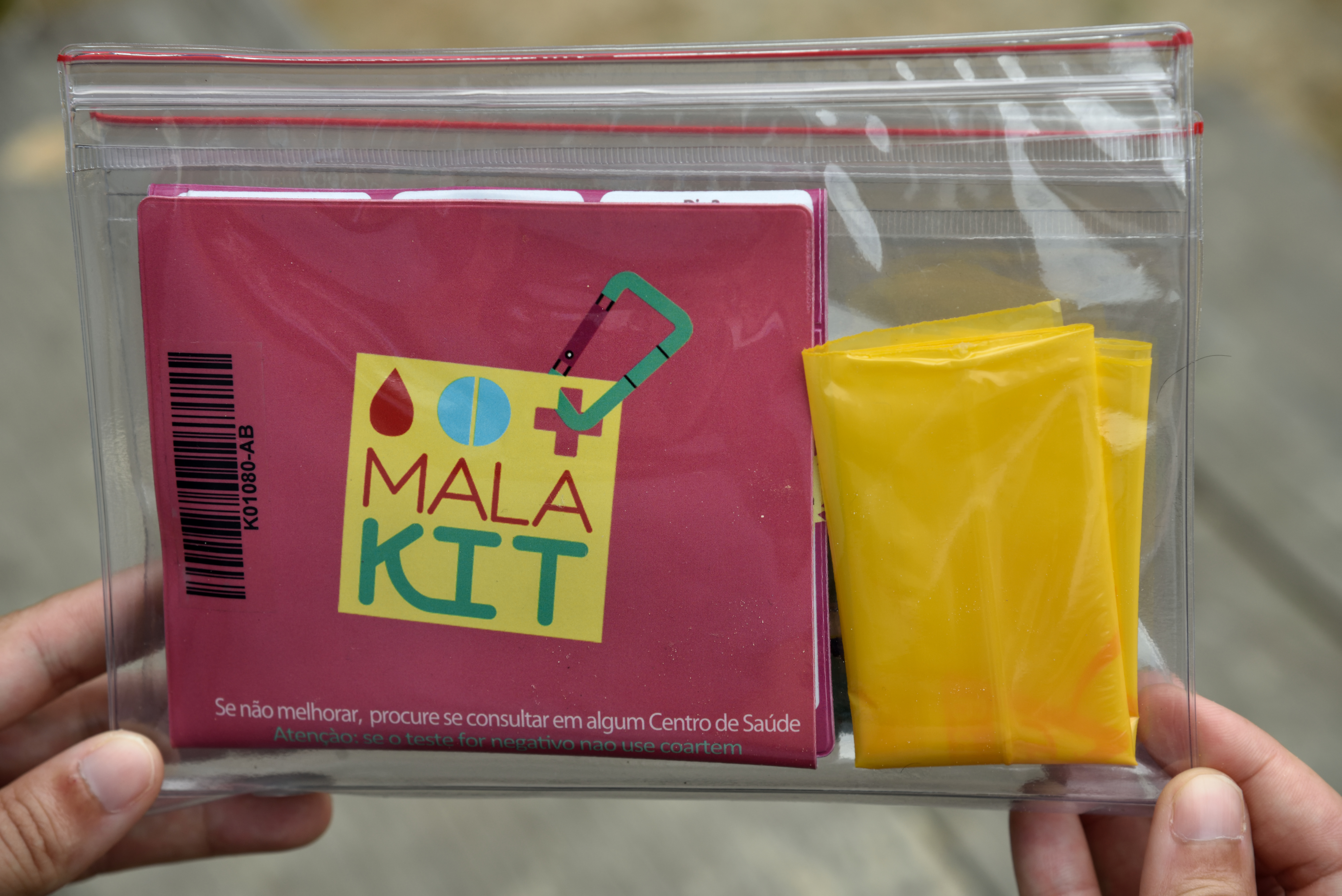

Supplement: Supplementary file 2 — Additional file 2. Malakit pouch. Picture of a malakit closed. [file 12936_2021_3748_MOESM2_ESM.jpg]

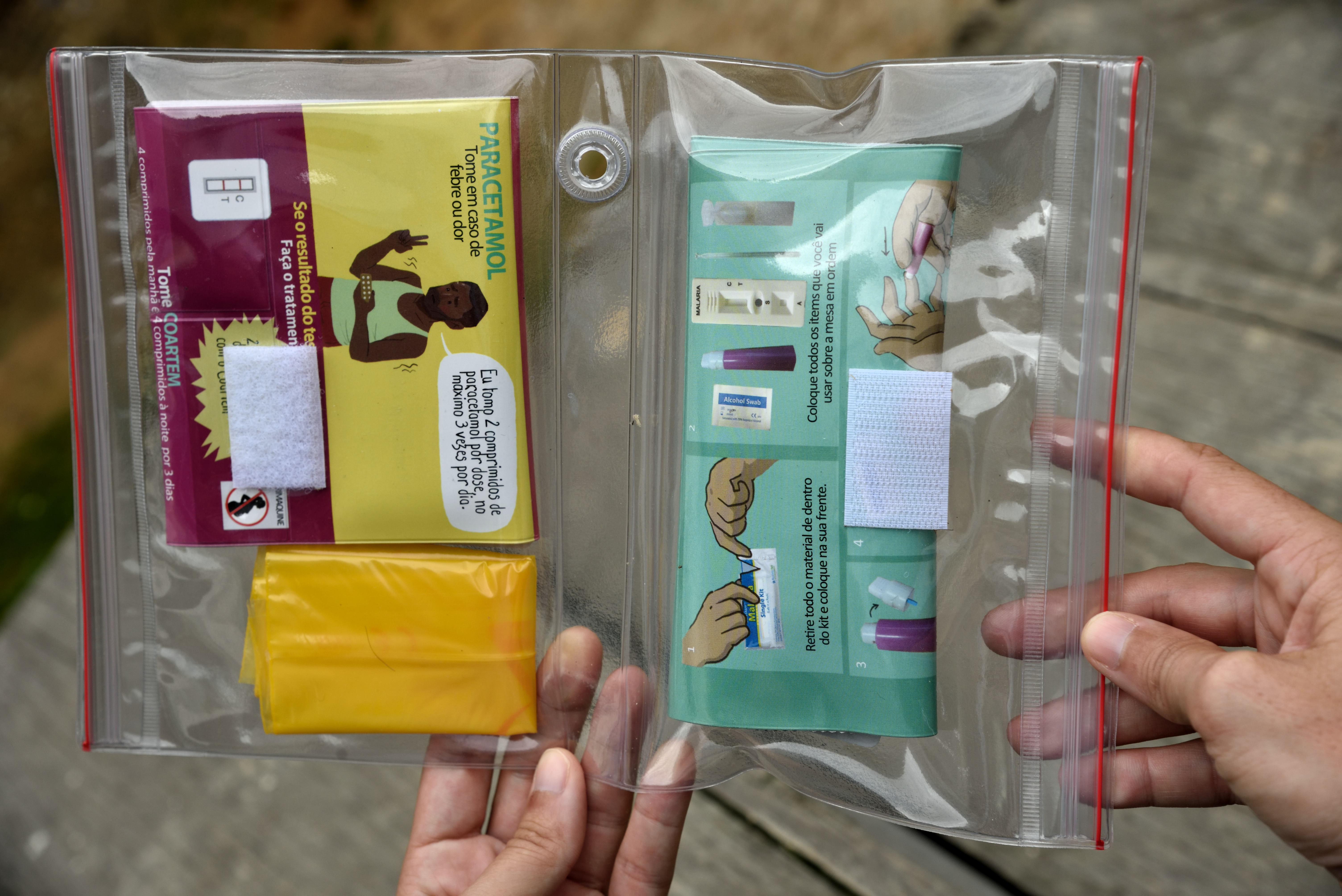

Supplement: Supplementary file 3 — Additional file 3. Malakit pouch opened. Picture of a malakit opened. [file 12936_2021_3748_MOESM3_ESM.jpg]
