# Supplementary material for: Setting-up a cross-border action-research project to control malaria in remote areas of the Amazon: describing the birth and milestones of a complex international project (Malakit)
Source: Malar J. 2021 May 11;20:216. doi: 10.1186/s12936-021-03748-5 (PMC8111981; doi:10.1186/s12936-021-03748-5)
Supplement: Supplementary file 4 — Additional file 4. RDT pocket of the malakit. Picture of the RDT pocket heads (illustrated instructions) and tails. [file 12936_2021_3748_MOESM4_ESM.pdf]

MALA KIT

Quando se sentir doente, o melhor é você se consultar em algum Centro de Saúde

Atenção: se o teste for negativo nao use coartem

1
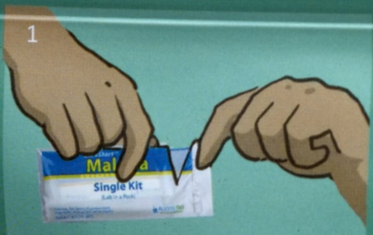

Retire todo o material de dentro do kit e coloque na sua frente.

2
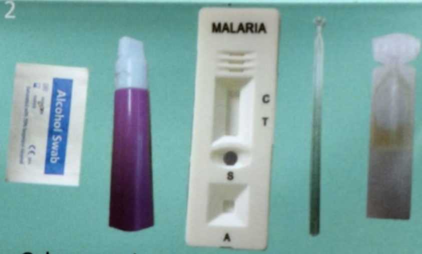

Coloque todos os items que você vai usar sobre a mesa em ordem

3
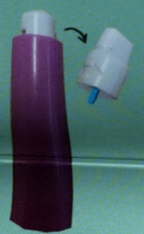

remova a tampa da lanceta

4
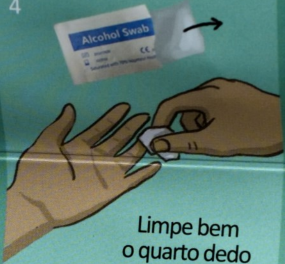

Limpe bem o quarto dedo com o alcool

5
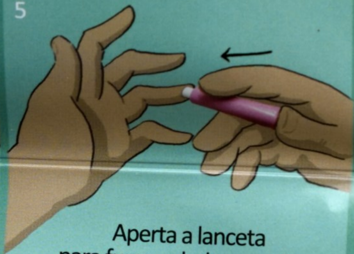

Aperta a lanceta para furar o dedo, ao soltar, a agulha volta por si só

6

Massageie o dedo até formar uma gota de sangue

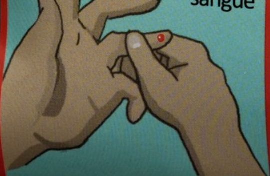

E préciso formar uma gota com o sangue

7
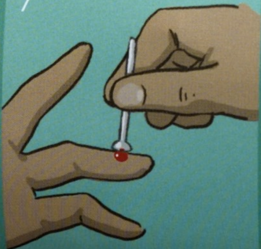

Pegue uma gota de sangue com a pipeta

8
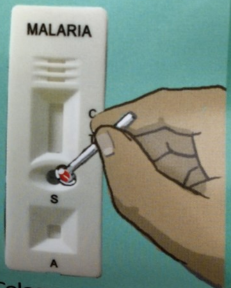

Coloque a gota no poço marcado S

9
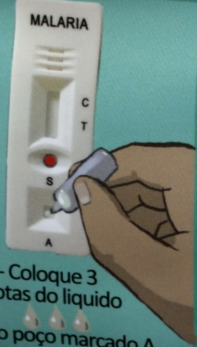

9 - Coloque 3 gotas do liquido no poço marcado A

10
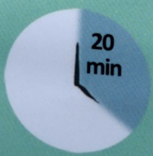

Espere **20 minutos** sobre uma superficie plana.

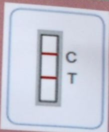

2 linhas = Positivo

Faça o tratamento contra a malària

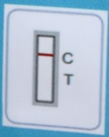

Linha C = Negativo

Não faça o tratamento contra a malària

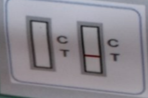

Nenhuma linha ou apenas uma linha no nivel da letra T = invàlido

refazer o teste

Tome paracétamol se você tiver febre ou dores

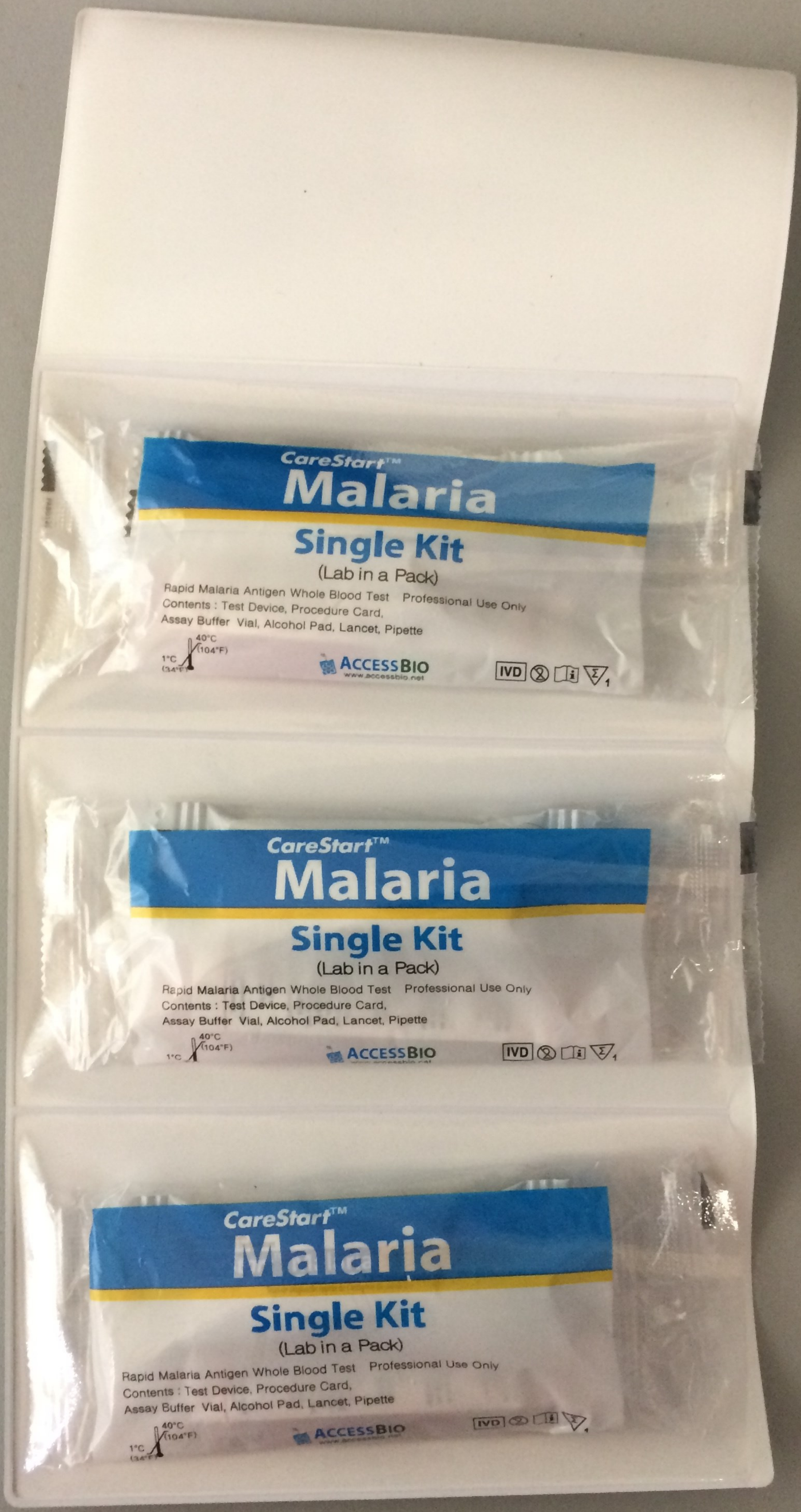
CareStart™
Malaria
Single Kit
(Lab in a Pack)
Rapid Malaria Antigen Whole Blood Test Professional Use Only
Contents : Test Device, Procedure Card,
Assay Buffer Vial, Alcohol Pad, Lancet, Pipette
40°C
(104°F)
1°C
(34°F)
ACCESSBIO
www.accessbio.net
IVD
CareStart™
Malaria
Single Kit
(Lab in a Pack)
Rapid Malaria Antigen Whole Blood Test Professional Use Only
Contents : Test Device, Procedure Card,
Assay Buffer Vial, Alcohol Pad, Lancet, Pipette
40°C
(104°F)
1°C
ACCESSBIO
IVD
CareStart™
Malaria
Single Kit
(Lab in a Pack)
Rapid Malaria Antigen Whole Blood Test Professional Use Only
Contents : Test Device, Procedure Card,
Assay Buffer Vial, Alcohol Pad, Lancet, Pipette
40°C
(104°F)
1°C
ACCESSBIO
IVD
