# Supplementary material for: Setting-up a cross-border action-research project to control malaria in remote areas of the Amazon: describing the birth and milestones of a complex international project (Malakit)
Source: Malar J. 2021 May 11;20:216. doi: 10.1186/s12936-021-03748-5 (PMC8111981; doi:10.1186/s12936-021-03748-5)

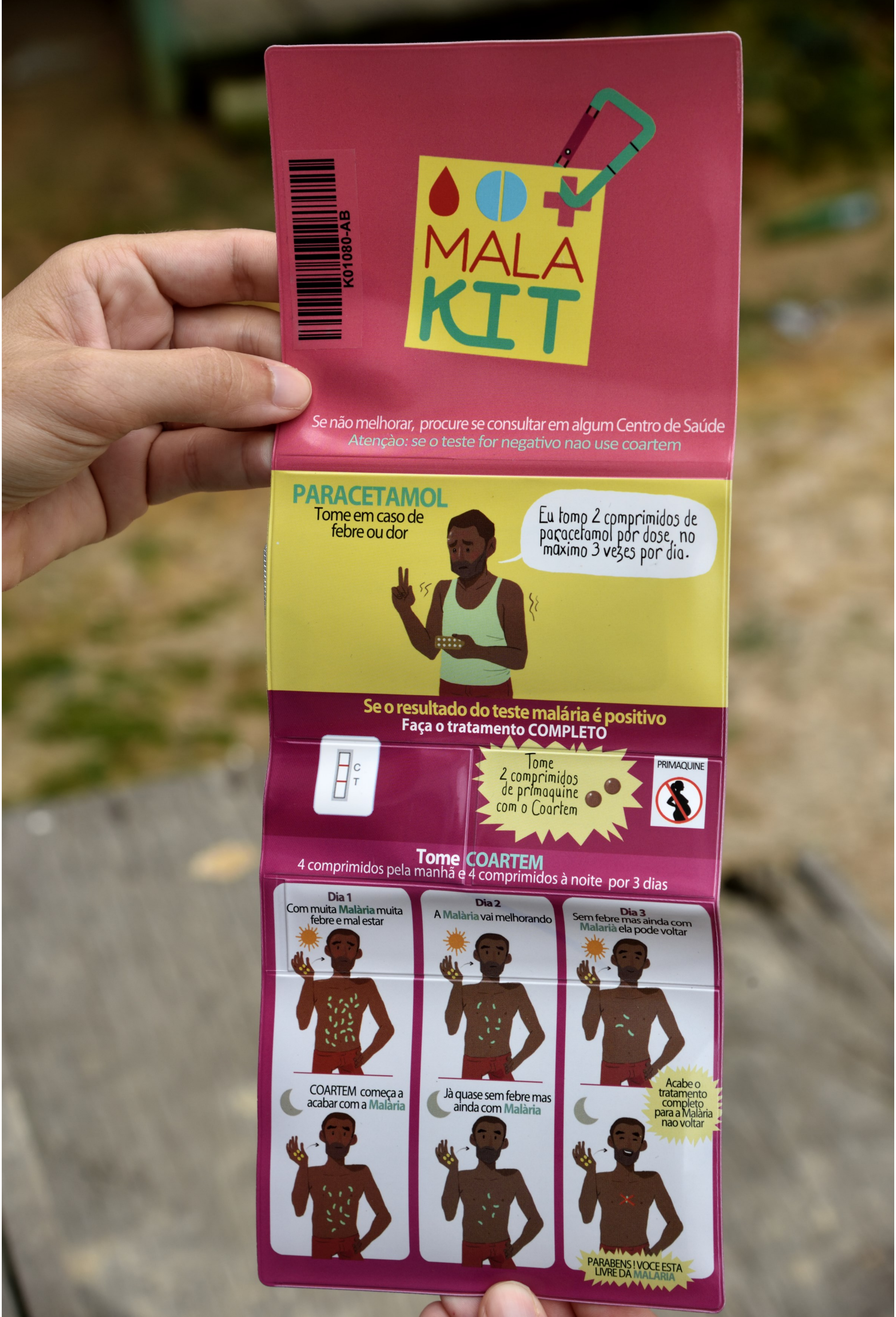
K01080-AB
MALA KIT
Se não melhorar, procure se consultar em algum Centro de Saúde
Atençào: se o teste for negativo nao use coartem
PARACETAMOL
Tome em caso de febre ou dor
Eu tomo 2 comprimidos de paracetamol por dose, no máximo 3 vezes por dia.
Se o resultado do teste malária é positivo
Faça o tratamento COMPLETO
C
T
Tome 2 comprimidos de primaquine com o Coartem
PRIMAQUINE
Tome COARTEM
4 comprimidos pela manhã e 4 comprimidos à noite por 3 dias
Dia 1
Com muita Malària muita febre e mal estar
COARTEM começa a acabar com a Malària
Dia 2
A Malària vai melhorando
Jà quase sem febre mas ainda com Malària
Dia 3
Sem febre mas ainda com Malarià ela pode voltar
Acabe o tratamento completo para a Malària nao voltar
PARABENS ! VOCE ESTA LIVRE DA MALARIA

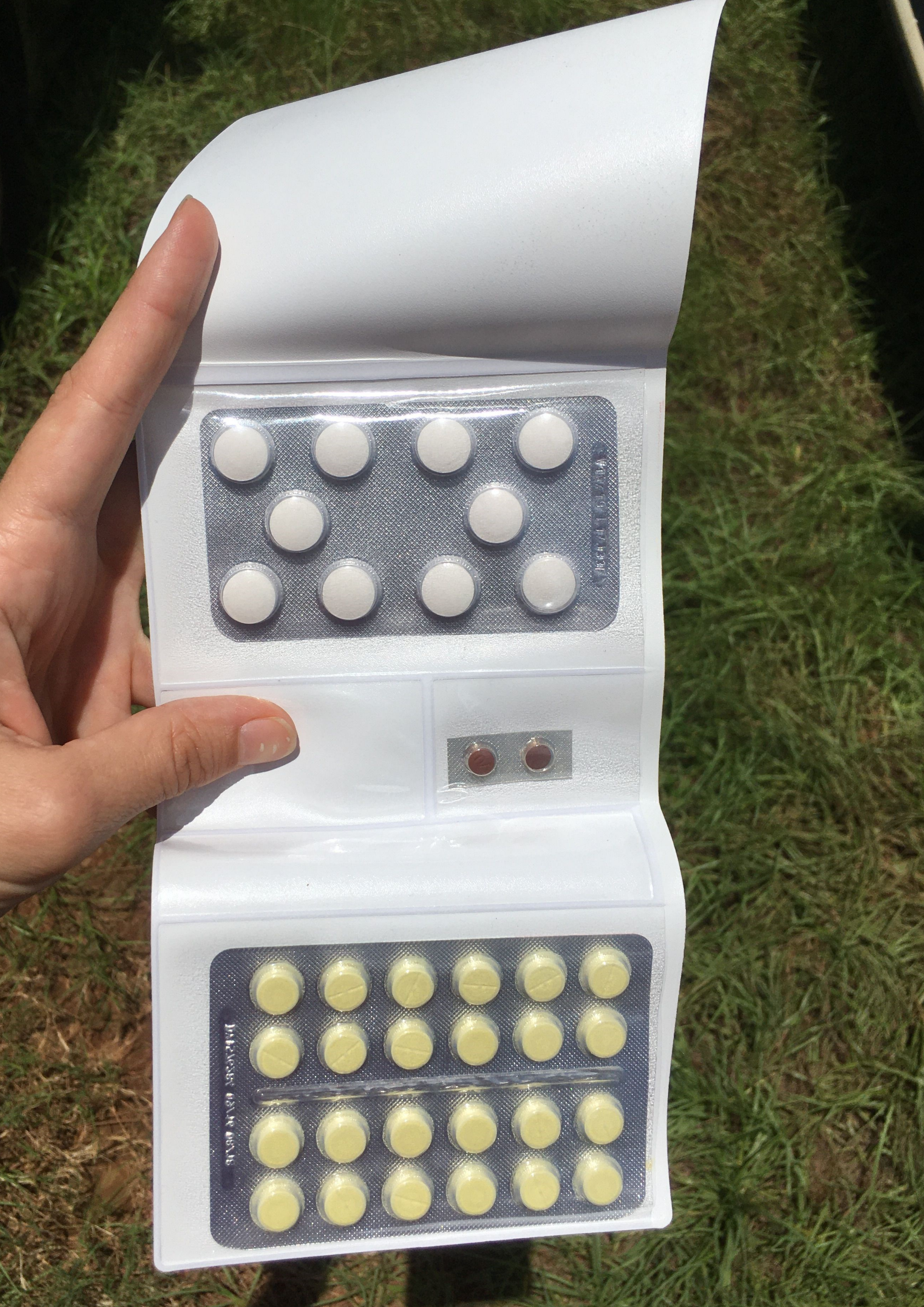

Supplement: Supplementary file 5 — Additional file 5. Treatment pocket of the malakit. Picture of the Treatment pocket heads (illustrated instructions) and tails. [file 12936_2021_3748_MOESM5_ESM.pdf]
