# Supplementary material for: Setting-up a cross-border action-research project to control malaria in remote areas of the Amazon: describing the birth and milestones of a complex international project (Malakit)
Source: Malar J. 2021 May 11;20:216. doi: 10.1186/s12936-021-03748-5 (PMC8111981; doi:10.1186/s12936-021-03748-5)
Supplement: Supplementary file 6 — Additional file 6. Malakit training course. Content of the training. [file 12936_2021_3748_MOESM6_ESM.pdf]

Malakit training course

The Malakit training course (undertaken after the course on malaria) for facilitators included:

- Context and objectives of the project
- Rationale and novel aspects of Malakit
- Ethics, rights of participants, anonymity and informed consent
- Deeper knowledge of malaria case management in Malakit context
- Geography of French Guiana
- Training of trainers
- Data collection: first visits and follow-up visits
- Kit assembly and stock management
- Use of digital tools for training, data collection and logistics
