# Supplementary material for: Setting-up a cross-border action-research project to control malaria in remote areas of the Amazon: describing the birth and milestones of a complex international project (Malakit)
Source: Malar J. 2021 May 11;20:216. doi: 10.1186/s12936-021-03748-5 (PMC8111981; doi:10.1186/s12936-021-03748-5)
Supplement: Supplementary file 7 — Additional file 7. Tools and contents for facilitator training. The different tools and their content used to train the facilitators in their tasks. [file 12936_2021_3748_MOESM7_ESM.pdf]

Tools and contents for facilitator training

- Facilitator material:
    - Information about the project including the baseline-endline study “Orpal”
    - Tasks of the facilitator
    - Participants’ rights as part of Good Clinical Practice
    - Information about the kit medication and RDT, important to know but not to share with participants
    - Wording and language elements for important messages
    - Answers to potential Frequently Asked Questions
- Guide for completing the questionnaire and paper forms by the facilitator
- Detailed instructions for the questionnaire administration and the filling of the paper forms.
- Kit assembly training tool: Sept-by-step pictured guide
- Illustrated memo of the order of the stages of training and enrollment :

Malakit: Estágios de inclusão

1. Recrutar no máximo 4 pessoas: verificas a idade e sua atividade na Guiana Francesa.	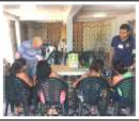
2. Explicar:<br>• um treinamento de uma hora<br>• um teste com picada (autoteste).<br>• assinar um formulário de consentimento.	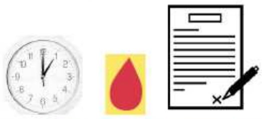
3. Mostrar o vídeo sobre o Malakit	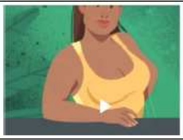
4. Mostrar o kit	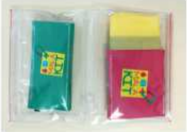
5. Falar com o participante sobre os seus conhecimentos e experiência em matéria de malária	Você já teve malária?<br>Quais são os sintomas da malária?
6. Mostrar e explicar os desenhos	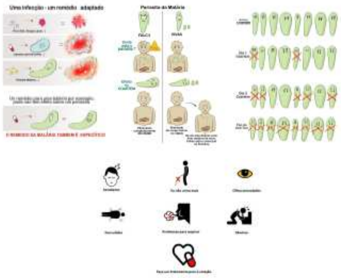

8. Mostrar o vídeo do kit (TDR)	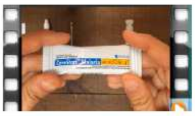
9. Mostrar a instruções no kit	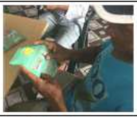
10. Parte pratica do teste (TDR)	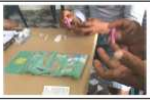
11. Durante o tempo de espera, explicar como fazer o tratamento	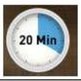 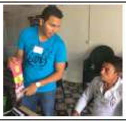
12. Apresentar a nota explicativa, preencher e mandar assinar os formulários	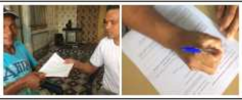
13. Questionário, colar as etiquetas e scanner e verificar leitura do teste (compreensão)	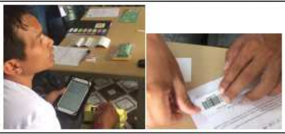
14. Dar na cartinha	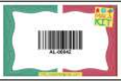
15. Entregar o kit e o mosquiteiro	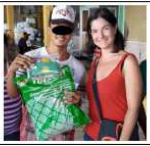
