# Supplementary material for: Setting-up a cross-border action-research project to control malaria in remote areas of the Amazon: describing the birth and milestones of a complex international project (Malakit)
Source: Malar J. 2021 May 11;20:216. doi: 10.1186/s12936-021-03748-5 (PMC8111981; doi:10.1186/s12936-021-03748-5)
Supplement: Supplementary file 10 — Additional file 10. Training posters. The drawings used by the facilitators to train the Malakit participants. [file 12936_2021_3748_MOESM10_ESM.pdf]

Uma infecção - um remédio adaptado

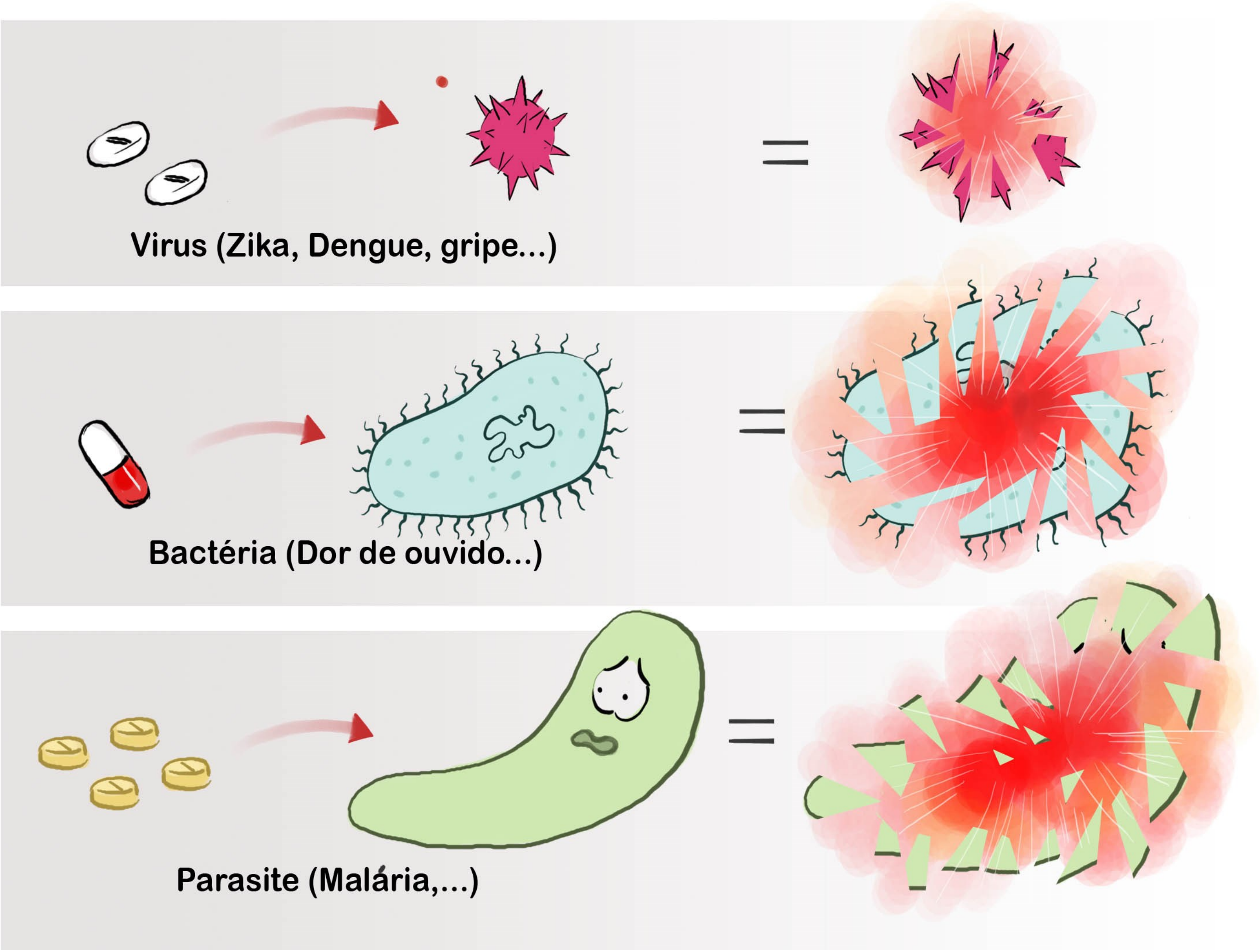

Un remédio para uma batéria por exemplo, pode nao tem efeito sobre um parasita.

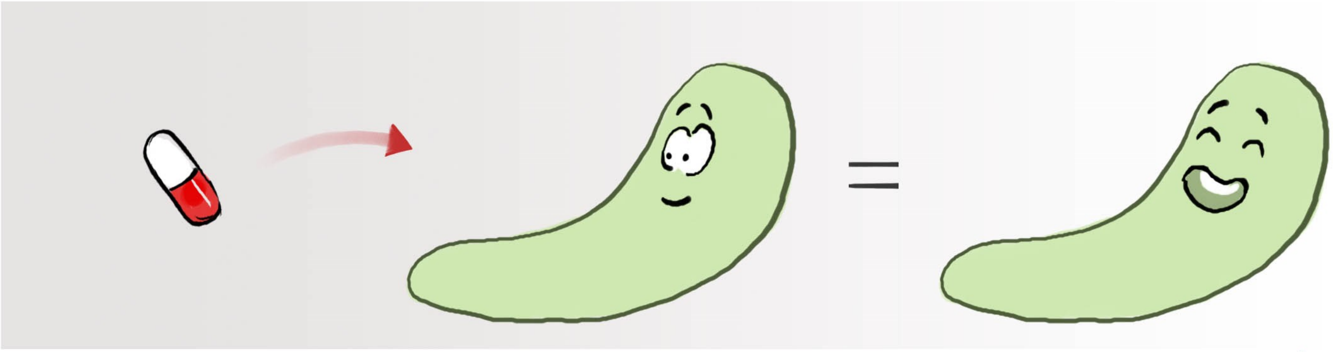

O REMEDIO DA MALÁRIA TAMBEM É ESPECÍFICO

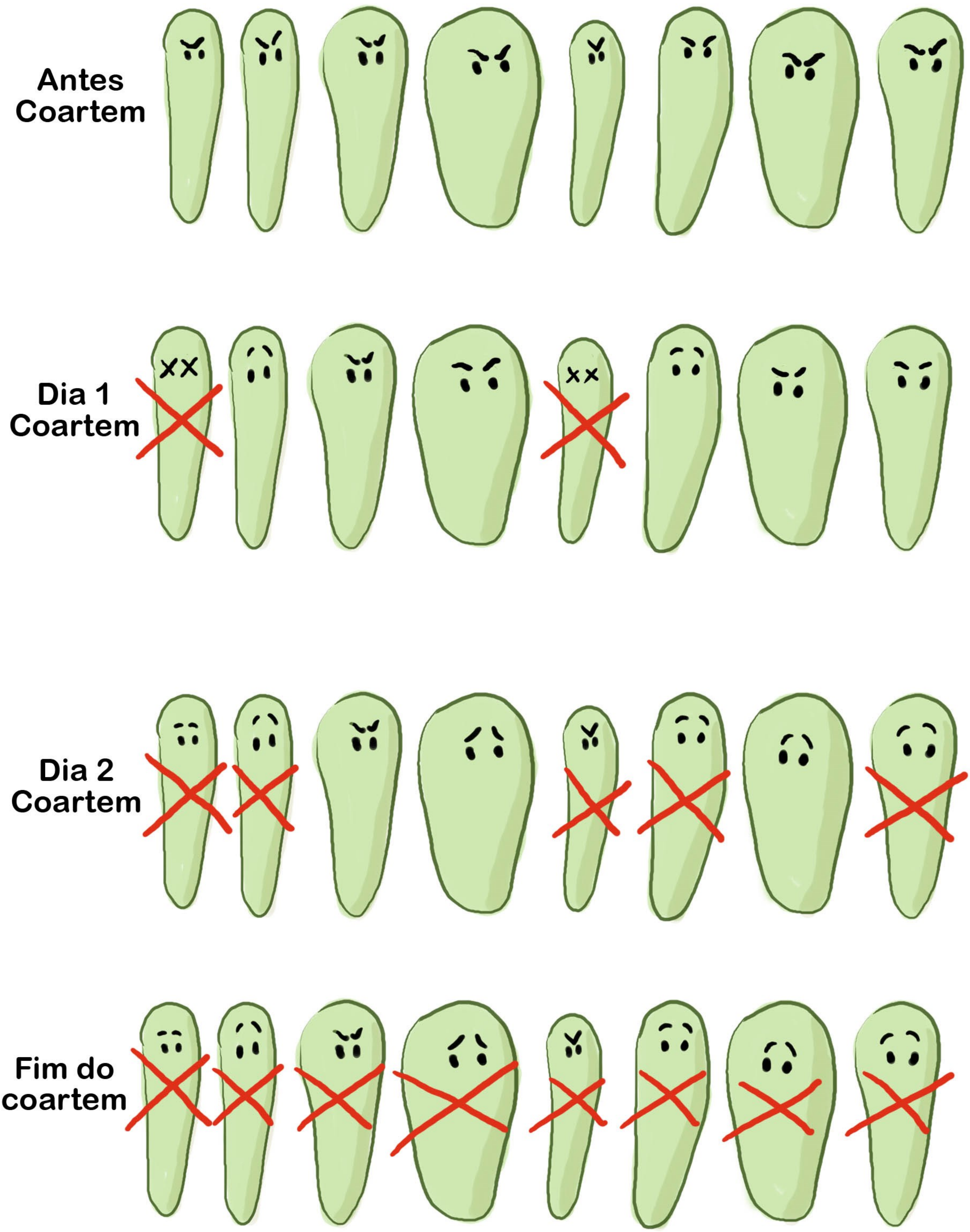
Antes
Coartem
Dia 1
Coartem
Dia 2
Coartem
Fim do
coartem

Parasita da Malária

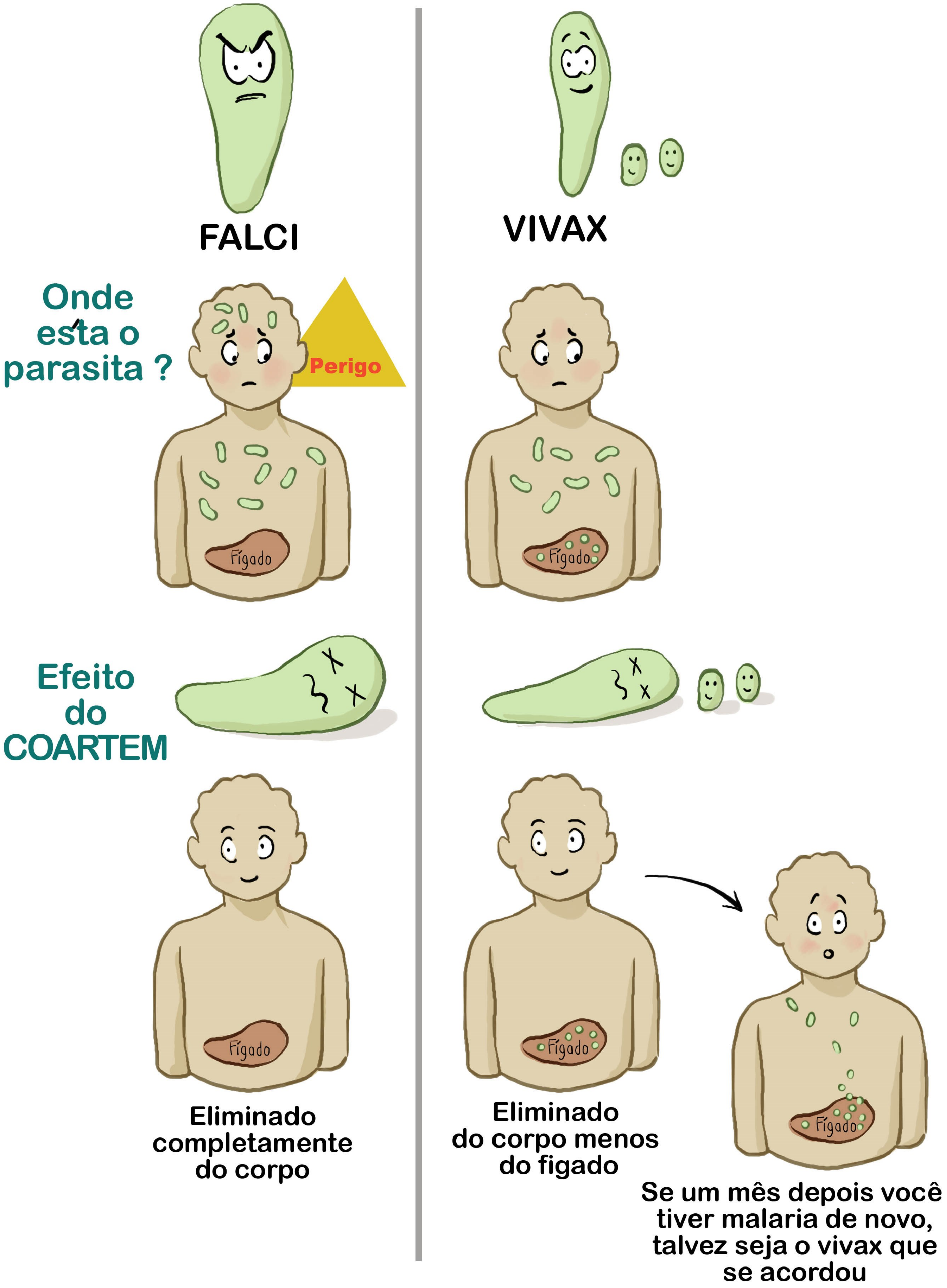

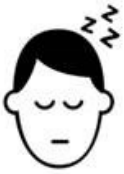

Sonolento

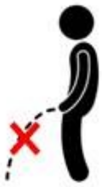

Eu não urino mais

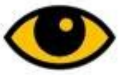

Olhos amarelados

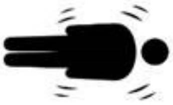

Convulsões

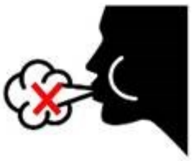

Problemas para respirar

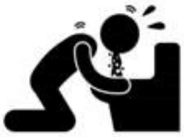

Vômitos

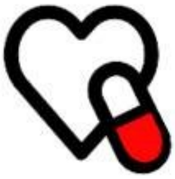

Faço um tratamento para o coração
